# Genetic Profiles of Ferroptosis in Malignant Brain Tumors and Off-Target Effects of Ferroptosis Induction

**DOI:** 10.3389/fonc.2021.783067

**Published:** 2021-12-01

**Authors:** Marc Dahlmanns, Eduard Yakubov, Jana Katharina Dahlmanns

**Affiliations:** ^1^ Institute for Physiology and Pathophysiology, Friedrich-Alexander-University Erlangen-Nuremberg, Erlangen, Germany; ^2^ Department of Neurosurgery, Paracelsus Medical University, Nuremberg, Germany; ^3^ Independent Researcher, Erlangen, Germany

**Keywords:** ferroptosis, neuroblastoma, glioblastoma, erastin, neuron, xCT, brain tumor therapy, off-target effects

## Abstract

Glioblastoma represents the most devastating form of human brain cancer, associated with a very poor survival rate of patients. Unfortunately, treatment options are currently limited and the gold standard pharmacological treatment with the chemotherapeutic drug temozolomide only slightly increases the survival rate. Experimental studies have shown that the efficiency of temozolomide can be improved by inducing ferroptosis – a recently discovered form of cell death, which is different from apoptosis, necrosis, or necroptosis and, which is characterized by lipid peroxidation and reactive oxygen species accumulation. Ferroptosis can also be activated to improve treatment of malignant stages of neuroblastoma, meningioma, and glioma. Due to their role in cancer treatment, ferroptosis-gene signatures have recently been evaluated for their ability to predict survival of patients. Despite positive effects during chemotherapy, the drugs used to induce ferroptosis – such as erastin and sorafenib – as well as genetic manipulation of key players in ferroptosis – such as the cystine-glutamate exchanger xCT and the glutathione peroxidase GPx4 – also impact neuronal function and cognitive capabilities. In this review, we give an update on ferroptosis in different brain tumors and summarize the impact of ferroptosis on healthy tissues.

## Introduction

Ferroptosis is as an iron-dependent form of cell death, which is different from previously known forms of cell death such as apoptosis, necrosis, or necroptosis. It is characterized by the accumulation of reactive oxygen species (ROS) and lipid peroxidation (1–3). After finding that activating ferroptosis in cancer cells of mice improved the effectiveness of temozolomide treatment – a first-line chemotherapeutic drug against glioblastoma (glioma WHO grade IV) (4, 5) – further investigations revealed the important role of ferroptosis also in human cancer patients.

Glioma is a type of primary brain tumor that is generated from glial cells in the central nervous system. These gliomas are classified by the WHO into low-grade glioma (WHO grade II) and high–grade glioma (WHO grade III/IV), where higher grading is associated with poorer prognosis (6). Ferroptosis represents an option to improve treatment for patients suffering especially from these more malignant tumors, including glioblastomas, because these are difficult to cure by radiation, resection, or pharmacological treatment alone. Especially because pharmacological treatment is affected by drug resistances (7).

Since the discovery of ferroptosis in 2012 (1) several key molecules have been identified, which are either directly integrated into the ferroptosis process or act as inducers. Current data about key players in ferroptosis and their role in glioma have been reviewed elsewhere (8, 9). The recently launched database ferrDB provides an overview of these regulators and markers in ferroptosis (10).

**Figure 1:**
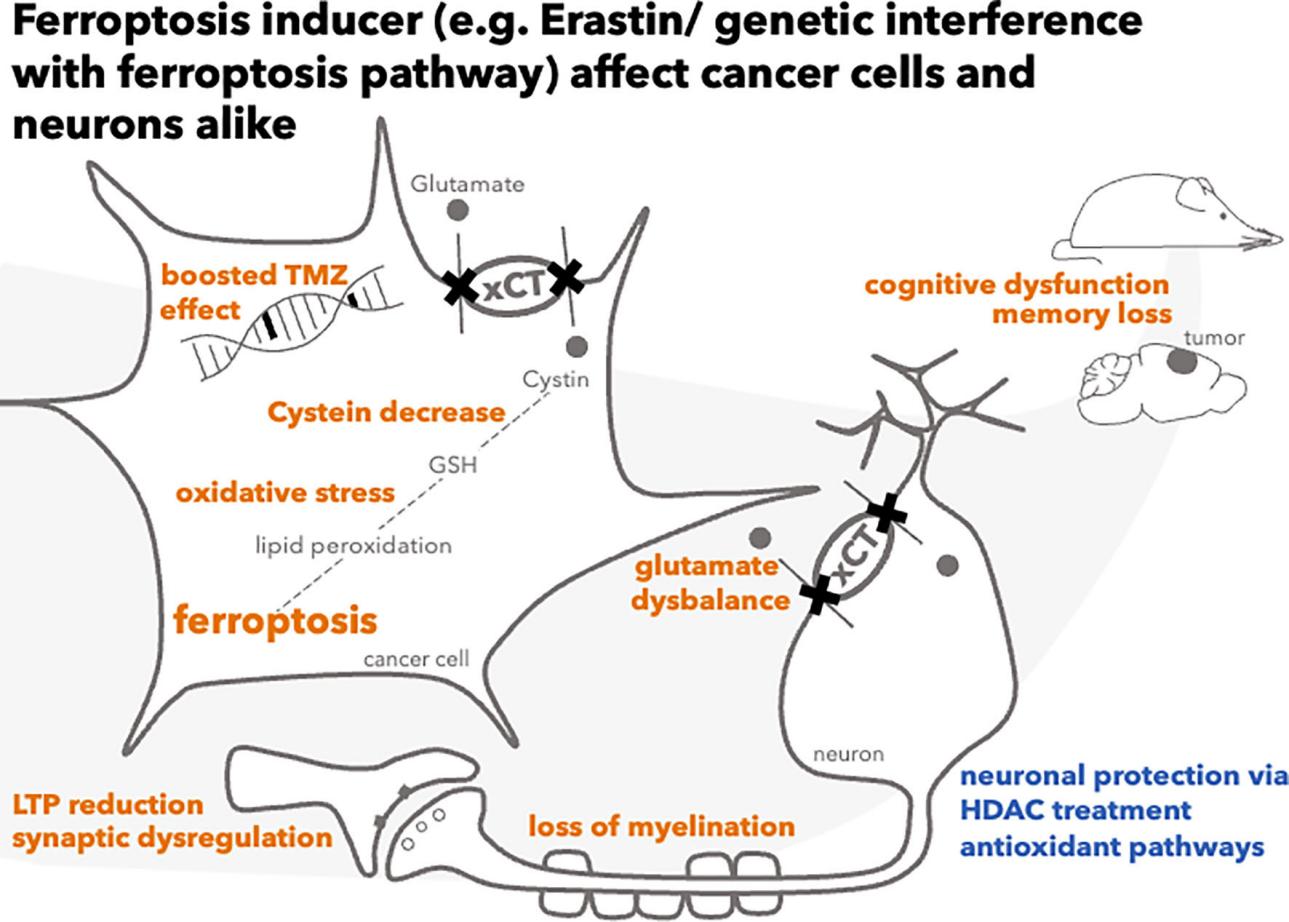
Consequences of ferroptosis induction in cancer cells and neurons.

This review provides an overview of ferroptosis in the therapy of various brain tumors with a focus on ferroptosis gene signatures, which have a strong translational value in predicting patients’ prognosis, and of the effects of ferroptosis induction in non-cancerous tissue that is also affected during treatment (**Figure 1**).

## Promising Findings on Ferroptosis Induction in Neuroblastoma and Meningioma

Expanding on the treatment boosting effects of ferroptosis induction in glioma, there are also promising findings in other types of cancer. Neuroblastoma is a highly relevant pediatric cancer in younger children (11), with limited treatment options and therapy resistance if occurring in its high–risk form (12). Induction of ferroptosis to limit tumor growth has been emerging as a striking new concept to treat neuroblastoma.

Ferroptosis can be induced by several small molecules [as reviewed elsewhere (8)] or by inhibition of the glutathione peroxidase GPx4 (13) and glutamate/cystine antiporter system $x_{c}^{-}$ (SLC7A11; also referred to as xCT) through the drugs erastin (1), sulfasalazine (14), or sorafenib (15), amongst others.

Recently, treatment with the steroidal lactone withaferin A was found to induce the nuclear factor erythroid 2–related factor 2 (Nrf2) pathway and to inactivate the GPx4 pathway, a duality making this strategy highly effective in treating both neuroblastoma cells and transplanted xenografts in mice (16). In this study the substance was targeted to the tumor site with nanoparticles, which reduces side-effects (17).

Chemosensitization to erastin–induced ferroptosis was also accomplished after knockdown of the iron exporter ferroportin in neuroblastoma SH-SY5Y cells (18).

In about 25% of neuroblastoma cases MYCN is amplified (19). In patient-derived xenografts of these cases, the xCT-driven antioxidant response after sulfasalazine application is increased compared to controls, which leads to an increase in ferroptosis and subsequently limited tumor growth (19). Further studies revealed that the transferrin receptor 1 was upregulated in response to such MYCN amplification, leading to increased GPx4 sensitivity and rendering neuroblastoma cells vulnerable to ferroptosis induction (20). In addition to this genetically mediated sensitization, the inhibition of PKCα stimulated ferroptosis and sensitized neuroblastoma stem cells to etoposide, which is particularly relevant given the central role of stem cells in conferring resistance to therapy (21). Neuroblastoma cell lines also express a very low level of ferritin heavy chain 1, whose reduction leads to a rise in ROS and a higher sensitivity to ferroptosis (22). In meningioma cell lines derived from patients covering WHO Grades I–III, the vulnerability to erastin-induced ferroptosis was increased both by a loss of neurofibromin and by a low level of E-cadherin. The expression of these proteins is driven by the myocyte enhancer factor 2C, making it a promising factor to manipulate during meningioma treatment (23, 24).

In summary, treatment of cancers such as neuroblastoma and meningioma in their advanced stages may be improved by exploiting the role of ferroptosis.

## Ferroptosis-Gene Signatures in Glioma

Gliomas represent a major form of brain cancer, divided into WHO grades I to IV with glioblastoma being the most devastating form of human brain cancer (6) because it is associated with a low survival, therapy resistance and limited treatment options (25). To overcome these obstacles, genetic studies based on large patient databases have examined the link between gene expression in glioma and overall survival in risk–stratified patient cohorts. In these studies, ferroptosis– and glioma–related genes of interest were identified by screening RNA sequencing data and associated clinical data. These gene–signatures constitute a risk-model, predicting the overall survival of the patients. To avoid overfitting, the models were each constructed in one database, e. g. Chinese Glioma Gene Atlas (CGGA), and validated using other databases, e. g. Repository for Molecular Brain in Neoplasia Data (REMBRANDT) or The Cancer Genome Atlas (TCGA) (26–32). The risk–models are shown and described in **Table 1**. The risk models that are based on the ferroptosis–related genes stratified glioma patients into a low–risk and high–risk cohort.

**Table 1:** Risk-models using ferroptosis-related genes and their predictive capabilities.

Study	Databases	Genes inside final gene signature	Gene function based on GO/KEGG	Correlation of the signature/risk model with:	What does the gene signature predict?
(26)	CGGA,	19 genes	Cell death, migration, and immune systems function → tumorigenesis and progression	WHO tumor grades, clinical/pathological tumor features	Overall survival
	TCGA, GSE16011,	SAT1, ATP5G3, HSPB1, FANCD2, HMGCR, CBS, GCLC, GCLM, CD44, ALOX12B, ALOX5AP, CISD1, NFE2L2, EMC2, ALOX4, DPP4, AKR1C2, LPCAT3 and NCOA4			
	REMBRANDT				
(27)	CGGA	25 genes	Responses to oxidative stress, nutrient level, and extracellular stimuli; pathways involve fatty acid synthesis, ferroptosis	1p/19q codeletion, IDH1 status, MGMT promoter methylation status, histology, age, WHO grading, PRS type	Overall survival
	TCGA	Protective: BAP1, GLS2, CISD1, PRNP, AKRIC3, TF, ACACA, ACSL6 and MAP3K5			
		Hazardous: CDKN1A, G6PD, HSPB1, LOX, STEP3, ACSL1, CP, HMOX1, CYBB, ANO6, PCBP1, PGD, AURKA, G3BP1 and TP53			
(31)	CGGA	12 genes (ferrDB-based)	Metabolic processes related to glutamate, immune systems response, and plasma membrane receptor complex	1p/19q codeletion, IDH1 status, MGMT promoter methylation status, radiation therapy	Overall survival
	TCGA	Protective: VDAC2, MAP3K5, DNAJB6, CHMP5			
		Hazardous: TP63, NFE2L2, MT3, LAMP2, HSPB1, FANCD2, ElF2AK4 and ARNTL			
(28)	CGGA	11 genes	Cancer progression by modulation of the immune system function	1p/19q non-codeletion, MGMT promoter methylation status, IDH status, recurrent and secondary tumors	Overall survival
	TCGA; GSE16011, REMBRANDT	Associated with a poor prognosis were a			
		-high expression of CD44, FANCD2, HSBP1, MT1G, NFE2L2 and SAT1 -low expression of AKR1C3, ALOX12, CRYAB, FADS2 and ZEB1			
(29)	REMBRANDT, CGGA-693, CGGA-325, TCGA	59 genes, metabolism of	Metabolism of iron, lipids, antioxidants, and energy	High risk scores: glioma WHO grade IV, IDH wildtype, no codeletion 1p/19q	Overall survival
		-Iron: FANCD2, NCOA4, TFRC, PHKG2, HSPB1, ACO1, FTH1, STEAP3, NFS1, IREB2, HMOX1 and MT1G -Lipid: ACSL4, AKR1C1-3, ALOX15, ALOX5, ALOX12, CARS, CBS, CISD1, CS, DPP4, GPX4, HMGCR, LPCAT3, FDFT1, ACSL3, PEPB1, ZEB1, SQLE, FADS2, ACSF2, PTGS2 and ACACA -Antioxidants: GCLC, SLC7A11, KEAP1, NQO1, ABCC1, CHAC1, GSS, GCLM and NFE2L2 -Energy: GLS2, SLC1A5, GOT1, G6PD, PGD and ATP5G3 Other genes: CD44, HSPB1, CRYABM, RPL8, SAT1, TP53, EMC2 and AIFM2			
(30)	TCGA, CGGA, REMBRANDT	15 Long non-coding RNAs:	–	Low-risk groups: Radiotherapy was effective	Overall survival
		SNAI3-AS1, GDNF-AS1, WDFY3-AS2, CPB2-AS1, WAC-AS1, SLC25A21-AS1, ARHGEF26-AS1, LINC00641, LINC00844, MIR155HG, MIR22HG, PVT1, SNHG18, PAXIP1-AS2, SBF2-AS1		High-risk group:	
				Unfavorable immunological situation	
(33)	Pubmed-reported ferroptosis-proteins, TCGA GBMLGG, CGGA	8 genes:	Lipid metabolism, carboxylic acid metabolism	IDH1_p.R132H (6/8), tumor purity (5/8), MGMT methylation (5/8),	-Overall survival -Progression-free survival
		ALOX5, CISD1, FTL, CD44, FANCD2, NFE2L2, SLC1A5, GOT1			

In high-risk cohorts, the median survival probabilities indicated by Kaplan-Meier curves were significantly decreased. The risk-score was often correlated with clinicopathological features such as the WHO grade or the O–6–Methylguanine–DNA Methyltransferase (MGMT) promotor methylation status, proving the suitability of ferroptosis–related gene expression pattern for patient outcome prediction. Interestingly, functional annotation of the ferroptosis–related genes in the risk–models revealed that often the immune system is involved: Investigation of RNA sequencing data from glioblastoma (TCGA) revealed that the expression levels of ferroptosis suppressors such as CD44, HSPB1 and SLC40A1 correlated with the degree of immunosuppression and were related to survival of patients (34). The expression of these suppressors could also be induced by acetaminophen (34).

This bioinformatics-based immunology-ferroptosis-link was substantiated by experimental data showing that glioma GL261 cells during their early ferroptotic stages (induced by RSL3) promoted the activation of dendritic cells, which indicates a vaccination-like effect of the tumor cells on the immune system (35). With this, a link between ferroptosis and immunological responses in the context of glioma is strongly suggested and awaits further experimental clarification.

In Wan et al. the relevance of a link between ferroptosis and metabolism in the context of glioma was suggested based on a database analysis (29). For tumors, their increased metabolic reliance on utilizing amino acids (36) and lipids (37) represents malignancy hallmarks of cancer in general. In glioma, the approach of developing an amino acid-risk score – alike the here-described panels concerning ferroptosis-related genes - revealed that the expression of genes involved in amino acid metabolism is important for glioma patients’ survival prognosis (38). In glioma with non-mutated isocitrate dehydrogenase (IDH), branched-chain amino acids such as leucine and isoleucine, and their catabolizing enzyme branched-chain amino acid transaminase 1 (BCAT1), are more expressed – in turn, BCAT1 knock-down in glioma cells reduces the viability of glioma cells (39). Underlining the role of ferroptosis during amino acid regulation in cancer, the induction of ferroptosis eventually inhibited transcription of BCAT2 and the direct inhibition of BCAT2 led to ferroptosis in target cells (40). Additionally, cysteine and glutamate represent important amino acids during ferroptosis induction (41), whose homeostasis is interrupted by blocking xCT to achieve cell death.

In general, cancer cell growth and development are reliant on an increased lipid usage (42, 43). Thus, interfering with these pathways by oxidation of the lipids may boost cancer therapy by exploiting ferroptosis (31, 37). Increased lipid peroxidation is the result of ferroptosis induction and eventually leads to ferroptotic cell death (1). As one of the proteins that was used to generate survival-predicting ferroptosis-related genes panels (**Table 1**), ACSL4, increases the content of omega-6 polyunsaturated fatty acids in cellular membranes and thus regulates how sensitive cells are to ferroptosis (44).

In one ferroptosis–signature panel, the data suggested that a risk–score built up by 19 ferroptosis genes was negatively correlated with the expression of MGMT, which confers resistance to temozolomide (26). However, many different mechanisms have been proposed to be contributing to temozolomide resistance in glioma (5, 45), which makes it difficult to assess their respective translational importance. Interestingly, not only coding RNA but also long non–coding RNA was shown to be predictive regarding overall survival (30). In addition to the common prediction of overall survival, one study was able to also accurately predict patients’ progression-free survival based on ferroptosis–related proteins (33).

While all presented risk models were capable of stratifying patients into high-risk and low-risk cohorts, the number of ferroptosis–related genes required to create the prognostic model substantially varies from 8 up to 59 included genes (**Table 1**). Redundancies of several genes between different risk models might indicate their general importance.

To evaluate if these genes are exclusively predictive of the outcome prognosis in glioma, we examined their role in comparable gene signature panels in other cancer types: A number of genes that are part of glioma risk–models (CARS, FANCD2, HMGCR, NCOA4 and SLC7A11) (46) and (AKR1C1, CARS1, CBS, CD44, CHAC1, DPP4, FANCD2, GOT1, HMGCR, SLC1A5, NCOA4 and STEAP3) (47) also accurately predicted patients’ prognosis in clear renal cell carcinoma. Similarly, survival probability in hepatocellular carcinoma was reliably predicted by a glioma prediction model (ACSL3, ACSL6, ACACA, G6PD, SLC1A5, SLC7A11 and VDAC2) (48) and by a risk model with a strong overlap with the genes in the glioma models (G6PD, HMOX1, LOX, SLC7A11, STMN1/Stathmin 1) (49). It is, however, unlikely that ferroptosis signatures are similar across all different types of cancer, which is exemplified by a study predicting breast cancer based on a completely different set of ferroptosis–related genes (50). It will be interesting to investigate the minimum number of expressed ferroptosis–related genes in a tumor for the patients’ outcome to benefit from ferroptosis induction and to investigate, how the gene expression is systematically distributed across different kinds of tumors.

## Ferroptosis In Healthy Neurons and Potential Side–Effects of Ferroptosis Induction

The functional property of cystine/glutamate exchanger xCT is the uptake of cystine and the extrusion of glutamate – a key molecule of neuronal function, whose homeostasis is key for proper signal transduction and cognitive behavior (39, 51, 52). Because another function of xCT is the stimulation of the antioxidative response of the cell, xCT–inhibitors can induce ferroptosis (1) **(** **Figure 1** **)**.

Given the promising preclinical finding of improved temozolomide (Temodal^®^, Temcad^®^) chemotherapy outcome through combination with xCT–inhibitory small molecules (4), it appears necessary to also investigate such drugs’ potential impact on other cells in the vicinity of the tumor tissue and in the whole body. In particular, diseases of the peripheral nervous system are known side–effects of some chemotherapeutic treatments (53), and also have been linked to ferroptosis (54).

Here, we take a closer look at the impact of xCT interference on neuronal and cognitive function (**Figure 1**)

An investigation of how the xCT inhibitors erastin and sorafenib affect cultured hippocampal neurons in their morphology and their vesicle pool size – a parameter tightly linked to neuronal function – has shown that such treatment could significantly disturb neuronal viability (55). In the hippocampus of xCT–deficient mice, long–term potentiation and long–term memory were impaired (56), which highlights the importance of xCT–driven glutamate homeostasis for cognition. Although a reduction of extracellular glutamate would be expected after xCT–inhibition or deletion, additional extracellular glutamate could not reverse this effect (56). In primary hippocampal cell cultures consisting of both, neurons and glia, extracellular amino acid profiling could not confirm a reduction, but rather an increase in extracellular glutamate after erastin–induced xCT inhibition, suggesting a complex regulatory interplay between different cell types of the brain (55).

Inhibition of xCT led to a myelination defect in organotypic cerebellar slices after a few days of treatment, showing that neuronal function is disturbed also on the axonal level (57).

On a behavioral level, xCT was linked to stress resilience in the ventral hippocampus, because alterations in the histone acetylation status increased xCT expression and in turn recruited other glutamate receptors to modulate glutamate homeostasis (58). Mice with intraperitoneal erastin injections developed iron depositions in several organs such as brain, kidney and spleen, mild cerebral infarction and epithelial changes in the duodenum (59).

Efforts to examine ferroptosis–inhibitory agents to protect against such adverse effects have demonstrated that hippocampal HT22 cells could be protected from ferroptosis with Ajudecunoid C – a chemical isolated from *Ajuga nipponensis* – *via* an activation of an antioxidant response element pathway (60), or with diphenylamine compounds (61). Similarly, spinal cord neurons have been protected from erastin–induced ferroptosis through LipoxinA4–induced activation of the Akt/Nrf2/HO–1 signaling axis (62), which represents a key player in the regulating of ferroptosis and also in glioma treatment (63–65). The impact of erastin on neuronal viability was further counteracted in primary cortical neurons and SH–SY5Y cells by the iron chelator deferoxamine (66). Despite ferroptosis being similar in neurons and cancer cells, class 1 histone deacetylase inhibitors (HDACs) treatment protected neurons from ferroptosis but augmented ferroptosis in HT1080 fibrosarcoma cells (67), thereby providing the best possible outcome. This promising finding now awaits its experimental evaluation in other cell types, for example in different glioma cell lines.

Ferroptosis can also be thwarted on other levels of the ferroptosis–inducing process, for example by selenium–mediated inhibition of the antioxidant glutathione peroxidase 4 (GPx4) (68), which is also implicated in the pathophysiology of glioblastoma (7, 69, 70b). Similar to xCT–deficient mice (56), conditional deletion of GPx4 in adult forebrain neurons resulted in impaired functions of memory and spatial learning (71), and its deletion from dopaminergic midbrain neurons increased anxious behavior (72). These examples from a list of several more ferroptosis–inhibitory agents demonstrate that such drugs, initially intended to counteract neurodegeneration, could also act as support during chemotherapy to protect healthy tissue.

In contrast to erastin, which remains a purely experimental substance, multi–kinase inhibitor and ferroptosis inductor sorafenib has entered human clinical trials that included assessment of neuropsychological effects during cancer therapy. Learning, memory, and executive functions suffered over the course of treatment (73). This is further supported by a study in rats that revealed neurochemical disturbances in the hippocampus during treatment with sorafenib (74). Although the histology of the hippocampus was unaffected in that study, treatment with sorafenib for 28 days strongly decreased levels of several key metabolites such as glutamate, GABA, serine, or choline, which were measured by nuclear magnetic resonance spectroscopy. In contrast, striatum and prefrontal cortex remained rather unaffected (74). In primary rat hippocampus cultures, high–performance liquid chromatography revealed that, already after 24 h of sorafenib treatment, levels of glutamate, serin, and alpha–aminobutyric acid were increased, and levels of glycine, cystine, and phosphoethanolamine were decreased (55). These data illustrate metabolic disturbances in response to sorafenib treatment, which may account for cognitive dysfunction.

In addition to emerging as possible side effects of ferroptosis pathway manipulation, cognitive impairment was also described as a glioblastoma symptom (75). Cognitive impairment often delays diagnosis and is associated with a reduced overall survival (75), which should be considered when assessing cognitive dysfunction as potential side effects of add–on drugs.

## Conclusion

The pharmacological therapy of malignant brain tumors is difficult, especially of late–stage glioma with its treatment resistance and recurrences. The novel idea of enhancing treatment outcome through ferroptosis induction continually gains attention. Recent data uncovered a link between ferroptosis–signatures in malignant glioma and overall survival, with many studies using expression of ferroptosis–related genes to accurately predict patients’ survival probability. Harnessing ferroptosis to improve tumor therapy will be an appealing approach also in malignant neuroblastoma and meningioma. But interfering with ferroptosis induction also has off–target effects, which may decrease the quality of life. Therefore, the increase in survival probability predicted by ferroptosis–gene-based risk models should be traded of against potential harm through ferroptosis–inducing add–on therapy. Ideally, patients should be screened for ferroptosis-related gene expression - based on a unified set of disease-relevant ferroptosis-related genes - and stratified into high-risk or low-risk cohorts to judge their individual clinical prospects. Future clinical trials may evaluate the benefits versus side effects of ferroptosis inducing cancer treatment enhancement for different patient groups.

In summary, ferroptosis induction is a hope yielding approach to enhance antitumor therapy but requires an intricate balance between attacking the tumor and preserving the different cell types of the healthy tissue.

## Authors Contributions

MD proposed the research. MD and JD both reviewed the literature and collected references. MD, EY, and JD wrote the manuscript and finalized the paper. All authors contributed to the article and approved the submitted manuscript.

## Conflict of Interest

The authors declare that the research was conducted in the absence of any commercial or financial relationships that could be construed as a potential conflict of interest.

## Publisher’s Note

All claims expressed in this article are solely those of the authors and do not necessarily represent those of their affiliated organizations, or those of the publisher, the editors and the reviewers. Any product that may be evaluated in this article, or claim that may be made by its manufacturer, is not guaranteed or endorsed by the publisher.

## Glossary

**Table:**

ACACA	Acetyl-CoA carboxylase 1	
ACO1	Aconitase 1	
ACSF2	Acyl-CoA Synthetase Family Member 2	
ACSL1/3/4/6	Acyl-CoA Synthetase Long Chain Family Member 1/4/6	
AKR1C1-3	Aldo-keto reductase family 1 member C3 1-3	
ANO6	Anoctamin 6	
AIFM2	Apoptosis Inducing Factor Mitochondria Associated 2	
ALOX12B	Arachidonate 12-Lipoxygenase 12R Type	
ALOX12	Arachidonate 12-Lipoxygenase 12S Type	
ALOX15	Arachidonate 15-lipoxygenase	
ALOX5	Arachidonate 5-Lipoxygenase	
ALOX5AP	Arachidonate 5-Lipoxygenase Activating Protein	
ARNTL	Aryl hydrocarbon receptor nuclear translocator-like protein 1	
ABCC1	ATP Binding Cassette Subfamily C Member 1	
ATP5G3	ATP Synthase Membrane Subunit C Locus 3	
AURKA	Aurora Kinase A	
BAP1	BRCA1 Associated Protein 1	
CISD1	CDGSH Iron Sulfur Domain 1	
CP	Ceruloplasmin	
CHAC1	ChaC Glutathione Specific Gamma-Glutamylcyclotransferase 1	
CHMP5	Charged multivesicular body protein 5	
CS	Citrate Synthase	
CD44	Cluster of differentiation 44	
CRYAB	Crystallin Alpha B	
CDKN1A	Cyclin Dependent Kinase Inhibitor 1A	
CBS	Cystathionine Beta-Synthase	
CARS	Cysteinyl-TRNA Synthetase 1	
SLC7A11	Cystine/Glutamine antiporter xCT or Solute Carrier Family 7 Member 11	
CYBB	Cytochrome b(-245) beta subunit	
DPP4	Dipeptidyl peptidase 4	
Hsp40	DnaJ Heat Shock Protein Family	
DNAJB6	Member B6	
EMC2	ER membrane protein complex subunit 2	
ElF2AK4	Eukaryotic translation initiation factor 2α kinase 4	
FANCD2	FA Complementation Group D2	
FDFT1	Farnesyl-Diphosphate Farnesyltransferase 1	
FADS2	Fatty acid desaturase 2	
FTL	Ferritin Light Chain	
G6PD	Glucose-6-phosphate dehydrogenase	
GCLC	Glutamate-Cysteine Ligase Catalytic Subunit	
GCLM	Glutamate-Cysteine Ligase Modifier Subunit	
GOT1	Glutamic-Oxaloacetic Transaminase 1	
GLS2	Glutaminase 2	
GPX4	Glutathione Peroxidase 4	
GSS	Glutathione Synthetase	
HSPB1	Heat shock protein beta-1	
HMOX1	Heme oxygenase 1 gene	
IREB2	Iron-responsive element-binding protein 2	
KEAP1	Kelch Like ECH Associated Protein 1	
LPCAT3	Lysophosphatidylcholine Acyltransferase 3	
LAMP2	Lysosome-associated membrane protein 2	
HMGCR	HMG-CoA reductase	
LOX	Lysyl Oxidase	
MT3	Metallothionein 3	
MT1G	Metallothionein-1G	
MAP3K5	Mitogen-activated protein kinase kinase kinase 5	
NQO1	NAD(P)H Quinone Dehydrogenase 1	
SLC1A5	Neutral amino acid transporter B(0)	
NFS1	NFS1 Cysteine Desulfurase	
NFE2L2	Nuclear factor-erythroid 2-related factor 2	
NCOA4	Nuclear Receptor Coactivator 4	
PEPB1	Phosphatidylethanolamine Binding Protein 1	
PGD	Phosphogluconate Dehydrogenase	
PHKG2	Phosphorylase Kinase Catalytic Subunit Gamma 2	
PRNP	Prion protein	
PTGS2	Prostaglandin-Endoperoxide Synthase 2	
G3BP1	Ras GTPase-activating protein-binding protein 1	
RB1	RB Transcriptional Corepressor 1	
RPL8	Ribosomal Protein L8	
STEAP3	Six-transmembrane epithelial antigen of the prostate 3	
SAT1	Spermidine/Spermine N1-Acetyltransferase 1	
SQLE	Squalene Epoxidase	
TFRC	Transferrin Receptor	
TP53	Tumor protein p53	
TP63	Tumor protein p63	
VDAC2	Voltage-dependent anion-selective channel protein 2	
ZEB1	Zinc Finger E-Box Binding Homeobox 1	
